#  The development of socio-motivational dependency from early to middle adolescence

**DOI:** 10.3389/fpsyg.2015.00194

**Published:** 2015-02-25

**Authors:** Danilo Jagenow, Diana Raufelder, Michael Eid

**Affiliations:** Methods and Evaluation Division, Department of Education and Psychology, Freie Universität BerlinBerlin, Germany

**Keywords:** socio-motivational dependency, person-oriented approach, latent transition analysis, teacher–student relationship, student–student relationship

## Abstract

Research on students’ motivation has shown that motivation can be enhanced or undermined by social factors. However, when interpreting such findings, interindividual differences, and intraindividual changes underlying students’ perception of peers and teachers as a source of motivation are often neglected. The aim of the present study was to complement our understanding of socio-motivational dependency by investigating differences in the development of students’ socio-motivational dependency from early to middle adolescence. Data from 1088 students on their perceptions of peers and teachers as positive motivators when students were in seventh and eighth grade were compared with data of the same sample 2 years later. Latent class analysis supported four different motivation types (MT): (1) teacher-dependent MT, (2) peer-dependent MT, (3) teacher-and-peer-dependent MT, and (4) teacher-and-peer-independent MT. Latent transition analysis revealed substantial changes between the groups. The perceived teacher influence on students’ academic motivation increased from early to middle adolescence. Divergent roles of peers and teachers on students’ academic motivation are discussed.

## INTRODUCTION

Although the association between social factors and students’ motivation has been studied extensively in the past (e.g, Juvonen and Wentzel, 1996; Wentzel, 1998, 2009b; Reeve, 2006; Deci and Ryan, 2008; Ladd et al., 2009; Wentzel et al., 2012), it is still unclear whether these relationships undergo important developmental changes across early to middle adolescence. Therefore, the present study employed a longitudinal design to better understand intraindividual changes regarding peers and teachers as sources of motivation.

Raufelder et al. (2013c) examined interindividual differences in how important teachers and peers are in shaping the level of students’ academic motivation. Within the school context, students’ motivation can be predominantly affected by peers’ motivation, learning behavior, and social support or teachers’ motivation and support or both (Wentzel, 2009a,b; Raufelder et al., 2013c). However, this was not true for all students because a considerable part of students reported that their motivation is not affected by teachers and peers. Research supported the concept of four different types of what is termed as socio-motivational (in-)dependency^1^: (1) a teacher-dependent motivation type (MT), (2) a peer-dependent MT, (3) a teacher-and-peer-dependent MT, (4) a teacher-and-peer-independent MT (Raufelder et al., 2013c; Hoferichter et al., 2014). The teacher-dependent MT is described as a student whose academic motivation is predominantly affected by teacher’s characteristics and perceived support, such as awareness of the student’s abilities or the teacher’s own engagement, motivation and interest in the subject. In contrast, the peer-dependent MT represents students whose motivation is predominantly affected by the school-based behavior of their peers, such as interests, motivation or the amount of effort they put into studying. In turn, the teacher-and-peer-dependent MT is a mixed type because academic motivation of such students is affected by both teachers’ and peers’ characteristics. Hoferichter et al. (2014) compared a sample of Canadian and German students and suggest that the teacher-and-student dependence presupposes the developing social competencies more than the other types. Finally, the teacher-and-peer-independent MT is unaffected by teachers’ and peers’ motivation, learning behavior, and support. (Jagenow et al., 2014) compared the various forms of socio-motivational dependency based on different school-related variables. Despite the assumption that the sources of motivation of the independent type lies in the individual themselves (i.e., intrinsic motivated) these students showed lowest level of academic motivation while lowest test anxiety values. The authors suggest that the independent type might at risk of school failure.

## LITERATURE REVIEW

Literature, investigating the link between relationships at school and motivation points to the existence of different forms of support teachers and peers can provide. Wentzel et al. (2010, 2012) distinguish between multiple classroom support: providing help, providing emotional support, creating a save environment, and communication of expectations and values. Raufelder et al. (2013c) interest was not only in support but also in students’ perceptions of teachers’ motivation for the subject, the awareness of students’ abilities or success with the material, and peers’ learning behavior. Students’ perception of these important classroom aspects is affecting most individuals’ motivation and achievement behavior. For example, when teachers show motivation for their subjects, students are expected to show more motivation as well, which is true for the subtypes teacher-dependent and teacher-and-peer-dependent MT (Raufelder et al., 2013c). However, it should be underlined that there are many students, whose motivation is unaffected by teachers and peers at all – the teacher-and-peer-independent MT.

Research has shown that the level of students’ academic motivation changes as they move through adolescence (Wentzel, 2009b). Namely, the motivation of many students decreases with each school year, due to changes both in social processing and in the school environment (Eccles and Midgley, 1990; Wigfield et al., 1998). Interestingly, students react in different ways to these changes: while some individuals start to resile from achievement situations and avoid such contexts whenever possible, others might not be negatively affected by the changes at all (Wigfield and Eccles, 2001). In other words, they show comparable levels of school achievement and motivation to learn throughout adolescence (Deci and Ryan, 2000) which seem to be independent of the teachers who instruct them or the classmates who surround them (Raufelder et al., 2013b).

A framework that helps to understand these divergent findings in adolescent motivation is ‘developmental contextualism’ (Lerner, 1986, 1991, 1998), that is, a theory of human ontogenetic development that focuses on the changing relations or coactions between the developing individual and his or her context. This theory argues that the development of the person-in-context is depicted as a function of dynamic processes embedded in multilevel interactions between a person and his or her contexts. This suggests that in the context of academic motivation, teacher–student relationships and student–student relationships might play a critical role in adolescence. In line with the work of Hamre and Pianta (2001), who applied developmental contextualism to better understand the importance of the teacher–student relationship for academic motivation, academic motivation can be understood to be one component of a dynamic process involving the interplay between the developing adolescent and his or her school context (i.e., teacher relationships, peer relationships). Taken together, the evidence of interindividual differences in students’ socio-motivational dependency and the contextual changes over time suggests that also intraindividual changes within the developing individual might take place.

The study by Raufelder et al. (2013c) is one of the few that examined the joint influence of teachers and peers on students’ academic motivation (see also Wentzel et al., 2010) using a person-oriented rather than a variable-oriented approach in this area of research. The variable-oriented approach focuses on purely additive effects of the variables of interest (Davidson et al., 2010) by ignoring interactions between the variables (Rosato and Baer, 2012; Raufelder et al., 2013d). Furrer and Skinner (2003) conducted a person-oriented design study and investigated the importance of children’s relatedness (e.g., feelings of acceptance, interpersonal support) to teachers and parents. The authors found that positive experiences in one relational context may buffer the impact of negative relational experiences in another context. This study also demonstrated an important advantage of the person-oriented approach, that is, it is designed to identify subgroups in the population that are characterized by a particular combination of values on a set of variables (Bergman and Magnusson, 1997; von Eye and Bergman, 2003). Furthermore, the person-oriented approach aims to group individuals into categories, where each contains individuals that are similar to each other and different from the individuals in other categories (Muthén and Muthén, 2000).

In the context of emotional and behavioral attitude toward school, the relationships that students have with their classroom peers have been repeatedly shown to play a critical role (e.g., Ryan, 2001; Furrer and Skinner, 2003; Zimmer-Gembeck et al., 2006; Ladd et al., 2009; Wentzel et al., 2010). For example, in a longitudinal study of adolescents, students who had better relationships with peers at school were more likely to display greater emotional engagement at school (Furrer and Skinner, 2003). Furthermore, the adolescents’ peer group context predicts the levels of school achievement and intrinsic school values (e.g., enjoyment, linking) after 1 year (Ryan, 2001). Ladd et al. (2009) argued that peer relationships promote students’ motivation by increasing student participation, and providing support and assistance, which increases students’ school engagement, as well as the overall levels of learning and academic competence.

Research on the development of social relationships during childhood and adolescence found that with the beginning of adolescence individuals spent a large part of their time in school and interact with their peers. Thus, it is likely that peers have an increasing impact on students’ school engagement as have teachers or parents (e.g., Csikszentmihalyi and Larson, 1984; Brown, 1990; Hymel et al., 1996; Rohrbeck, 2003; Levitt, 2005). One possible explanation for this behavior is that young adolescents have strong needs for social identity and spending time with peers satisfies these needs stronger than spending time with family members (Csikszentmihalyi et al., 1977; Youniss, 1980). Moreover, this period of time in one’s life is accompanied by significant changes in the nature of relationships with peers (e.g., Dubow et al., 1991; Larson and Richards, 1991). For example, in a study with children and adolescents, participants perceived parents and friends as equally supportive for the ages 9–15 but for the years 16–18 friends’ support exceeded parents’ support (Bokhorst et al., 2010). Dubow et al. (1991) found that changes from third through fifth graders in the received peer support were closely related to changes in their school adjustment 2 years later, which reflects the growing influence of peers during early adolescence. Other findings suggest, that adolescents whose friends do well in school or have a positive attitude toward school show fewer academic problems (e.g., disengagement) than those whose friends are less academically engaged (Crosnoe et al., 2003). Overall, it is reasonable that during adolescence peers play an increasingly stronger role in shaping one’s academic motivation because they satisfy one’s needs for close relationships, emotional, or behavioral support, as well as influence one’s attitude toward school.

In contrast, the quality of relationships with adults declines from the age of 12 to 18 (e.g., Kenny et al., 2013). In spite of the fact that teachers are the primary adult figures in the academic context, support from teachers declines from the age of 12 to 18 (Bokhorst et al., 2010). Moreover, the strength of the association between teachers’ support and students’ motivation decreases with each year passing (Goodenow, 1993). A critical time point is when students move from elementary to secondary school (Eccles et al., 1993), at which point the decline in the teacher–student relationship quality coincides with a growing need for close emotional relationships with adults from outside of the home environment (Midgley et al., 1989; Raufelder, 2007; Raufelder et al., 2013a).

There is a large body of research focusing on the link between teacher–student relationships and important academic outcome (e.g., Baker, 2006; Zimmer-Gembeck et al., 2006; Davidson et al., 2010; Wentzel et al., 2010; Hughes, 2012). Positive teacher–student relationships are associated with higher academic skills (e.g., Baker, 2006), classroom motivation (Wentzel, 1998), and social engagement (Gest et al., 2005). For example, middle school students tend to be more academically active in the classroom when they get positive feedback from their teachers (Skinner and Belmont, 1993), when they believe that their teachers care about them (Roeser et al., 1996), or when they are well-liked by their teachers (Wentzel and Asher, 1995). However, only few longitudinal studies have investigated the developmental changes of those associations (e.g., Skinner et al., 1998; Murdock et al., 2000; Hamre and Pianta, 2001; Furrer and Skinner, 2003). Hence, very little is known about the changes in the importance of these relationships for students’ motivation and school achievements after grade eighth. “Although we generally know more about these relationships at the elementary level, there is good reason to think that they are especially critical during secondary school” (Gehlbach et al., 2012, p. 692).

## RESEARCH OBJECTIVES AND HYPOTHESES

The aim of the current study was twofold. The first aim was to examine interindividual differences in adolescent students’ socio-motivational dependency. Specifically, we tested whether the four types of socio-motivational dependency found in seventh and eighth graders (Raufelder et al., 2013c) can be identified within the same cohort approximately 2 years later. The second aim of the present study was to describe the intraindividual development of students’ socio-motivational dependency from early to middle adolescence. We examined this age group because students’ motivation declines after the transition to secondary school and continues to do so for the first 3 years of high school (Harter, 1996), reaching its nadir in the ninth grade (Eccles and Wigfield, 1998).

Based on these preliminary studies, the empirical findings outlined above the present study examined the following two hypotheses:

### Hypothesis 1

(A) The same four types of socio-motivational dependency that were found in grade seven and eight (Raufelder et al., 2013c) can be identified in grade nine and 10. (B) More specifically, based on findings that peers become more important while adults become less important agents during adolescence (e.g., Juvonen and Wentzel, 1996), the size of the teacher-dependent MT group should decrease and the size of the peer-dependent MT should increase from early to middle adolescence.

### Hypothesis 2

Based on Lerner’s contextualism considering changing relations or coactions between the developing individual and his or her context over time (Lerner, 1986, 1991, 1998), it was hypothesized that individuals will vary in their socio-motivational dependency from early to middle adolescence. In particular, we assumed that the three socially dependent MTs would show higher fluctuation rates compared to the teacher-and-peer-independent MT.

## MATERIALS AND METHODS

### PARTICIPANTS

We initially examined 1088 participants aged 12–15 years (mean age = 13.7 years, SD = 0.53 years; 53.9% girls), who were sampled from a group of seventh- and eighth-grade students from 23 randomly chosen secondary schools in a suburban, predominantly middle-class community in Brandenburg (Germany). At the second time point, ∼2 years later, 783 students (mean age = 15.3 years, SD = 0.50 years; 53.5% girls) of the initial sample remained in the study (72.0% retention rate; sex, age, and MT at the first occasion of measurement were no predictors of drop-out.). Data on ethnicity was not collected due to low ethnic diversity in Brandenburg and socio-economic information was not gathered due to laws in Germany prohibiting the collection of such data via a third party. The study was approved by the Department of Education, Youth and Sports of Brandenburg. Consent was obtained from parents, and assent was provided by the participants. Parents and students were informed that the survey would be voluntarily, anonymous and confidential.

### PROCEDURE AND MEASURES

At the beginning of the German school year, self-report measures of students’ perception of peers and teachers as positive motivators were collected during class time on two consecutive days. For this purpose, two subscales from the Relationship and Motivation Scale (REMO; Raufelder et al., 2013b) were used: (1) ‘Teachers as positive motivators’ (TPM, six items; e.g., “If the teacher is really interested in the topic, I am interested as well”; internal consistency at the first time point (T1): Cronbach’s α = 0.78; internal consistency at the second time point (T2): Cronbach’s α = 0.78), (2) ‘Peers as positive motivators’ (PPM, nine items; e.g., “If my friends want to do better at school, I also want to do better”; internal consistency at T1: Cronbach’s α = 0.80; internal consistency at T2: Cronbach’s α = 0.83). These two subscales assess the positive influence of teachers and peers on academic motivation, respectively. In both subscales, responses are measured on a 4-point Likert scale ranging from 1 (*strongly disagree*) to 4 (*strongly agree*). The same measures were collected again using identical testing procedures after approximately 2 years.

### ANALYSIS

#### Latent class analysis on the second occasion of measurement

We employed a latent class approach to identify different groups of socio-motivational dependency at T2. Our analyses are based on the latent class analysis (LCA) that led Raufelder et al. (2013c) to identify four MT at the first occasion of measurement. In their analysis parcels from the PPM and TPM items were created and dichotomized. Therefore, prior to conducting LCA, parcels were create from the nine items of the REMO subscale PPM and from the six items of the REMO subscale TPM determined by the initial factor analysis of the REMO validation study (Raufelder et al., 2013b). Little et al. (2002) list three reasons that parceling can be advantageous over using the original items: (1) estimating large numbers of items is likely to result in spurious correlations, (2) subsets of items from a large item pool will likely share specific sources of variance that may not be of primary interest, and (3) solutions from item-level data are less likely to yield stable solutions than solutions from parcels of items. Based on these considerations as well as the unidimensionality of the Peer-REMO subscales (P-REMO), items of PPM were randomly assigned to parcels. In contrast, the items of TPM were assigned to parcels based on a factor analysis, as the Teacher-REMO subscales (T-REMO) are not unidimensional.

Using LCA, participants can be grouped into classes, in which individuals are assumed to have identical patterns of solution probabilities. For analyzing T2 data several models, where each is differentiated by the number of latent classes, were compared to determine which model best fits the observed data. Using an iterative process, we initially chose a two-class solution and increased the number of classes incrementally until a good fit was achieved. Statistical model fit criteria were employed to determine the optimal number of classes. The statistical criteria used to guide this process were the lowest Akaike Information Criteria (AIC; Akaike, 1973), the lowest Bayesian Information Criteria (BIC; Schwarz, 1978), and the lowest sample-size adjusted BIC (a. BIC). Values of these criteria are useful to compare the fit of one model with the fit of other models. Additionally, the Bootstrap Likelihood Ratio Test (BLRT; McLachlan and Peel, 2000) was conducted to test the statistical significant benefit of a model (*p* < 0.05). The BLRT compares the fit of a model with ‘g’ latent classes versus that with ‘g minus one’ latent classes (H0). In a Monte Carlo simulation, the BLRT was demonstrated to be a consistent and robust indicator of the presence of additional latent classes (Nylund et al., 2007).

#### Latent transition analysis

To investigate individuals’ transition between latent classes over time, we employed latent transition analyses (LTA), which combines LCA with autoregressive modeling (specifically Markov models; Langeheine and Van de Pol, 1990), where the latter describes transitions among the classes associated with time passing. Several models were compared to determine which model corresponds best to the observed data. The models differed in their restrictions: the fully restricted model assumes that: (a) conditional probability and (b) class size are the same for each of the classes across time. In contrast, the unrestricted model makes no assumptions about equality of the measurement parameters across the classes and time. Finally, the semi-restricted model involves an assumption that conditional probabilities are invariant but allows the class sizes to be estimated freely across time.

Using an iterative process, we started with a two-class solution for the two occasions of measurement and increased the number of classes incrementally. Additional classes were added until a good fit was achieved. Statistical model fit criteria were used to determine the optimal restriction and number of classes. The statistical criteria used to guide this process were the lowest AIC, BIC, and sample-size adjusted BIC.

To account for missing data, models were estimated with full information maximum likelihood (FIML). All of the models in the present study were analyzed using the statistical software Mplus 7.11 (Muthén and Muthén, 1999–2012).

## RESULTS

### LCA FOR THE SECOND OCCASION OF MEASUREMENT

**FIGURE 1:**
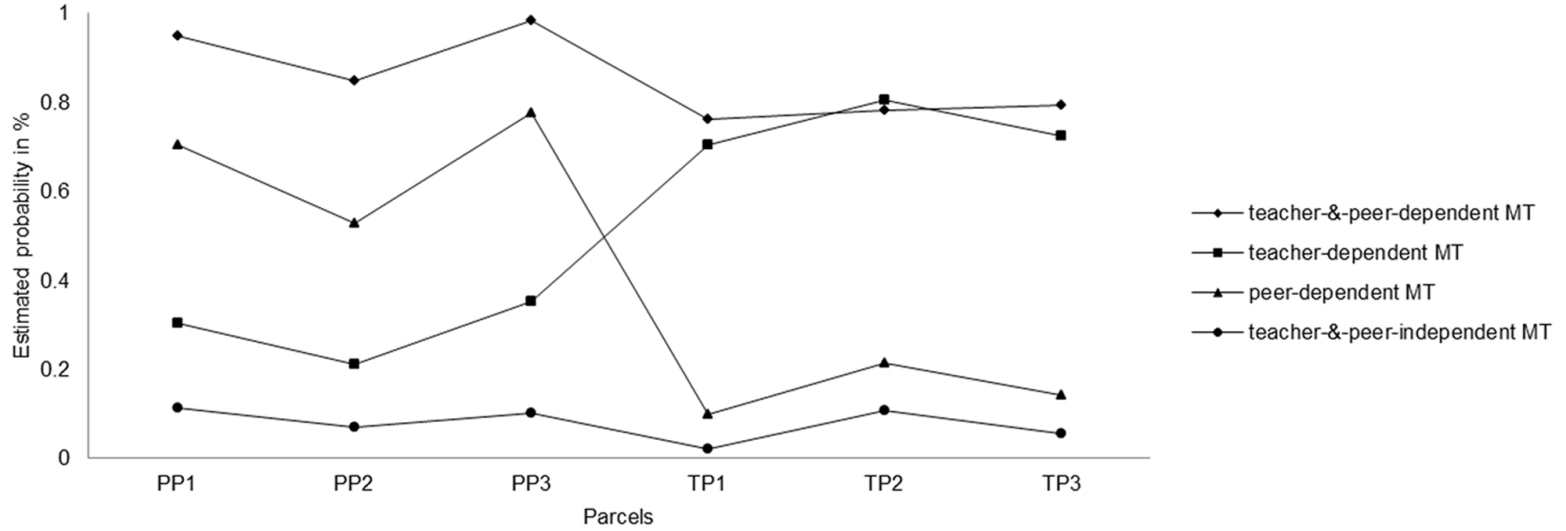
**Latent Class Analysis of socio-motivational dependency for the second occasion of measurement.** *X*-axis shows three “peers as motivators” parcels (PP1–PP3) and three “teacher as motivators” parcels (TP1–TP3) included in the model analyses. *Y*-axis shows probability of agreement with the clusters.

**Table 1** shows model-fit results for the LCA at T2 for the two through five class models. According to the AIC, BIC, and sample-size adjusted BIC (lowest values) the 4-class solution (model 3) had the best fit to our data. Furthermore, the BLRT indicated that model 4 is not superior to model 3. The classification quality of the model was satisfactory (entropy = 0.68). The entropy ranges from zero to one with higher values indicating a better class separation. To our knowledge, there is no simulation study that allows to define clear boundaries of entropy. The literature often refers to values around 0.60 as moderate (e.g., Vermunt, 2010), but there is no clear rule. **Figure 1** shows the estimated conditional class-specific probabilities to agree with the underlying indicator variables. Membership of the 4-class solution was as follows: 17.7% teacher-and-peer-dependent MT (74 girls and 64 boys), 19.8% teacher-dependent MT (83 girls and 72 boys), 35.2% peer-dependent MT, (148 girls and 129 boys), and 27.2% teacher-and-peer-independent MT (114 girls and 99 boys).

**Table 1:** Model fit results for latent class analysis (LCA) for the second occasion of measurement.

	Statistical fit criteria			Bootstrap Likelihood Ratio Test
	AIC	BIC	a. BIC	
(1) Model: 2 classes	6248.939	6310.581	6269.297	0.000
(2) Model: 3 classes	6096.016	6190.850	6127.336	0.000
(3) Model: 4 classes	**6002.511**	**6130.537**	**6044.793**	0.000
(4) Model: 5 classes	6010.158	6161.376	6053.402	0.105

AIC, Akaike Information Criteria; BIC, Bayesian Information Criteria; a. BIC, sample-size adjusted Bayesian Information Criteria; BLRT, Bootstrap Likelihood Ratio Test. Statistical fit criteria of the superior model are bold.

Compared to the class membership from the first occasion of measurement (see Raufelder et al., 2013c), the teacher-and-peer-dependent MT decreased by 10.1 percentage points (PPs), whereas the teacher-dependent MT increased by 10.3 PP. The groups of peer-dependent MT (increase by 0.9 PP) and independent MT (decrease by 0.9 PP) remained relatively stable.

### LTA FOR BOTH OCCASION OF MEASUREMENT

**Table 2** shows the model fit criteria for the LTA for the two through five class models and the three model restrictions. According to the AIC, BIC, and sample-size adjusted BIC (lowest values) the semi-restricted model involving a 4-class solution showed the best fit to our data. Results indicated measurement invariance (equality of conditional probabilities) from T1 to T2. Moreover, a change in class sizes had to be allowed. The classification quality of the model was satisfactory (entropy = 0.64).

**Table 2:** Statistical fit results for latent transition models (T1 and T2).

Number Of classes	Fully restricted			Semi restricted			Unrestricted		
	AIC	BIC	a. BIC	AIC	BIC	a. BIC	AIC	BIC	a. BIC
2	13840.5	13910.6	13866.1	13734.2	13809.2	13761.6	13720.5	13855.6	13769.8
3	13439.2	13549.3	13479.4	13292.7	13422.8	13340.2	13289.7	13509.8	13370.0
4	13242.9	13393.0	13297.7	**13050.3**	**13245.4**	**13121.5**	13051.4	13366.6	13166.4
5	13164.4	13504.6	13288.6	13069.1	13489.4	13222.6	13063.7	13634.0	13272.0

*AIC, Akaike Information Criteria; BIC, Bayesian Information Criteria; a. BIC, sample-size adjusted Bayesian Information Criteria. Statistical fit criteria of the superior model are bold*.

**Table 3** shows latent transition probabilities based on the estimated model^2^. In the following paragraph, the most vital results are described in more detail. The probabilities of staying in the same latent class at T1 and T2 are as follows: 0.68 for the independent MT, 0.58 for the peer-dependent MT, and 0.47 for the teacher-and-peer- dependent and the teacher-dependent MT, respectively. Furthermore, if you were a teacher-dependent MT at T1, the probability of becoming at T2 an independent MT is 0.40. If you were a teacher-and-peer-dependent MT at T1, the probability of becoming at T2 a peer-dependent MT is 0.28 and a teacher-dependent MT is 0.23. All other transition probabilities were below 0.20.

**Table 3:** Latent transition probabilities from T1 to T2.

	Latent class T2			
Latent class T1	Teacher and peer-dependent	Peer-dependent	Independent	Teacher-dependent
Teacher and peer-dependent	**0.47**	0.28	0.03	0.23
Peer-dependent	0.13	**0.58**	0.21	0.08
Independent	0.02	0.19	**0.68**	0.11
Teacher-dependent	0.10	0.03	0.40	**0.47**

*Probabilities of being in the same class at both occasions of measurement are bold*.

**Table 4** shows an absolute amount of stability and change types from T1 to T2, based on model’s estimated posterior probabilities. For example, 7.4% of the sample was a teacher-and-peer-dependent MT at T1 and became peer-dependent MT at T2; 6% of the sample was teacher-and-peer-dependent MT at T1 and became teacher-dependent MT at T2. 3% of the sample that became teacher-dependent MT at T2 was of peer-dependent MT at T1 and 2.5% of independent MT at T1.

**Table 4:** Transition probabilities.

Latent class pattern		
T1 class	T2 class	% of sample
1	1	12.5
1	2	7.4
1	3	0.9
1	4	6.0
2	1	4.6
2	2	21.0
2	3	7.4
2	4	3.0
3	1	0.5
3	2	4.5
3	3	15.8
3	4	2.5
4	1	1.6
4	2	0.4
4	3	5.6
4	4	6.6

*1 = teacher- and peer- dependent; 2 = peer-dependent; 3 = independent; 4 = teacher-dependent. Last row shows the proportion of individuals being in a latent class at T1 and T2*.

## DISCUSSION

The purpose of the present study was to extend our understanding of socio-motivational dependency by identifying interindividual differences and intraindividual changes over time in adolescent students’ socio-motivational dependency, following a person-oriented approach. In a first step, LCA was used to investigate interindividual differences in adolescents’ perception of peers and teachers as a source of motivation in ninth and tenth graders. In a second step, LTA were employed to investigate intraindividual changes in this perception from 7th and 8th to 9th and 10th grade.

In accordance with Hypothesis 1A, the same four types of socio-motivational dependency revealed in grade seven and eight by Raufelder et al. (2013c) could be identified 2 years later: (1) a teacher-and-peer-dependent MT, (2) a teacher-dependent MT, (3) a peer-dependent MT, (4) a teacher-and-peer-independent MT. These findings demonstrate presence of important interindividual differences in students’ socio-motivational dependency through adolescence, therefore underlining the validity of the typology itself.

What is important, the proportions of the sample constituting each type changed over time. However, the number of individuals that remained in the same class (55.9%) was slightly higher than those who changed to another type. In particular, the number of students who are teacher-dependent increased, whereas the group of the teacher-and-peer-dependent MT became smaller over time. In other words, Hypothesis 1B that peers become more important agents during adolescence was not confirmed. There are different explanations for this result. First, peers are still important for students’ academic motivation but their influence decreases. Second, the institutional relevance of teachers become more important in ninth or tenth grade, that is, when students’ final examines are closer. The teacher is critical when it comes to appraisal of achievement. This finding is in contrasts to those of Goodenow (1993), who found that the importance of the teachers in influencing motivation decreases through adolescence. Third, the teacher-and-peer-dependent group is larger than the teacher-dependent group and even a small transition probability from the former to the latter group might result in a large increase in the teacher-dependent group. Taken together, the increase in the size of the teacher-dependent MT group suggests an ambivalent role of the teacher–student relationship in middle adolescence.

Nevertheless, the peer-dependent MT group was the largest in size at the second occasion of measurement, which is consistent with the expectation that peers play an important role during adolescence in students’ academic motivation (Savin-Williams and Berndt, 1990; Fend, 1998; Brown and Theobald, 1999; Cook et al., 2007). Furthermore, the group of the independent MT did not change in size over time. This finding is important because other research suggest that students of this type seem to have no interest in school (i.e., they show the lowest scores in intrinsic motivation, achievement drive, and performance-approach goals; Jagenow et al., 2014), and, therefore, might be at high risk of school failure. However, the results underline the relevance of the typology and the considerations of strong interindividual differences, as some students show a socio-motivational dependency, while others show constant socio-motivational independence.

The results of the LTA demonstrated a substantial amount of transition across all classes with the smallest amount of mover in the independent MT. This finding is in line with Lerner’s developmental contextualism (Lerner, 1986, 1991, 1998) and confirms our Hypothesis 2. Intraindividual changes in the developing adolescence indicate contextual changes in the individuals’ school setting. However, it is still unclear why these changes are relevant for some students and for others not. Predictors of the different transition patterns might help to further enhance our understanding. For example, in future research the use of covariates might help to explain changes from one group to another.

For those students who were teacher-and-peer-dependent at the first occasion of measurement and became peer-only-dependent at the second occasion of measurement, the teachers influence the students’ motivation lesson students’ motivation. The reason might be that teachers’ support declines over the years (Bokhorst et al., 2010) and that this subgroup of students are sensible for such changes in the teacher–student relationship. However, almost the same transition probability was found for those becoming teacher-only-dependent from teacher-and-peer-dependent, suggesting that peers also can become less important for one’s academic motivation in the tested time period. Similarly, peers became less influential for some of the peer-dependent MT individuals, who subsequently switched to the independent MT. One explanation is that even though peer groups become increasingly important through adolescence (e.g., Csikszentmihalyi and Larson, 1984), this might not be the case for academic motivation. More research is needed to investigate the subgroups more closely. A high transition probability of becoming an independent MT was also found for those individuals who were previously of a teacher-dependent MT. However, this does not mean that the absolute change over time between the two groups is huge. 5.6% of the sample was teacher-dependent at the first occasion of measurement and independent at the second occasion of measurement (see **Table 4**). The finding of low probability of changing from an independent MT to a teacher-and-peer-dependent MT and vice versa is intuitively correct: a student whose motivation is affected by sources other than peers or teachers is unlikely to be substantially influenced by both approximately 2 years later.

Investigation of interindividual differences and intraindividual developmental changes in motivation typologies might facilitate the creation of programs and teaching activities that would support students within the school system based on their social needs in motivation. The typology employed by Raufelder et al. (2013c) important differences in the bidirectional interactions between students and their learning environment (Wigfield and Eccles, 2001). Education curricula and teacher training should take into account these differences in students’ motivation styles and how they develop across time. Such an approach would result in a more effective support of each student and accommodating their individual motivation profile. This typology helps to understand the differences in socio-motivational dependency, for example, why and how students interact with their social environment at school in their own specific way and at their own specific pace. In general, in school environments, students are expected to learn and behave uniformly, and students who do not follow these rigid expectations are typically viewed as maladjusted; while, as our research has shown, they just might have different motivational needs.

Our findings suggest that the motivational types should not be regarded or used as fixed labels because such an approach would inhibit an ability to see one’s unique potential in this respect, which might very likely change across time. The discussed typology underlines the fact that individuals tend to be socially motivated in various ways, and that a specific form of socio-motivational dependency exhibited by a student might substantially change over time.

The present study complements our understanding of interindividual differences and intraindividual changes in adolescents’ socio-motivational dependency. First, we investigated how the influence of teachers and peers on students’ academic motivation changes across adolescence, because little is known about these relations after grade eight. Second, we studied the nature of this development by using latent transition analysis. This approach enabled us to describe transitions across different MT that occur over time.

Despite these strengths, the limitations of the present study should be discussed. The current results are limited in their sole reliance on self-report measures. In future studies, teacher-reports should be used in combination with students’ self-reports to provide an additional source of evidence in examining differences in students’ academic motivation. Notably, studies that included teacher- and self-reports in research on student motivation have reported low levels of concordance between information provided by these sources (Skinner and Belmont, 1993). Furthermore, the longitudinal results should be interpreted considering variations due to changes in students’ motivation over the school year, as the data collection at the first time point was at the beginning of the German school year, whereas the data collection at the second time point was at the end of the German school year (see Gehlbach et al., 2012). Moreover, short-time dynamics in peer-relationships that might influence individuals’ motivation are not considered in the present study. Further research is necessary to determine personal factors and dispositional motivational characteristics of those individuals. Covariates can be included in LTA models to describe conditions of transition from one to another group.

In conclusion, the current study provided important novel findings regarding students’ socio-motivational dependency: across adolescence, for some students peers seem to have a decreasing influence on academic motivation, whereas for others this influence increases within this time period. A similar pattern characterizes the influence exerted by teachers on student academic motivation: for some individuals, teachers become more important in the time period of interest, while for others they do not. Although the magnitude of transition across different MTs was high, the number of students who are teacher-dependent increased and the number of those teacher-and-peer-dependents decreased over time, suggesting that teachers’ influence for students’ academic motivation might *de facto* increase across early to middle adolescence, possibly due to their importance for grades obtained in the final exams. Finally, there is an almost stable group of students with socio-motivational independency whose motivational sources should be examined in future studies. Overall, the current findings underline the need of focusing on interindividual differences in adolescents’ motivation, which should be considered in daily school life.

## Conflict of Interest Statement

The authors declare that the research was conducted in the absence of any commercial or financial relationships that could be construed as a potential conflict of interest.

## Footnotes

1 To clarify, Raufelder et al. (2013c) use the term “dependency” referring to motivation and thus differently, than the term is used in the context of learning-theoretical perspectives.

2 Sex was included as a covariate in the model but was not a significant predictor for transition probabilities from the first to the second occasion of measurement. For better presentation we report the model without covariates only.
